# Effect of *Bacillus subtilis* BS-Z15 metabolite mycosubtilin on body weight gain in mice

**DOI:** 10.3389/frmbi.2024.1301857

**Published:** 2024-03-13

**Authors:** Jun Yang, Jing-Jing Zhao, Jun-Qi Yue, Ming-Yue Ma, Huan Liu, Jia-Yi Chen, Xi-Yuan Cao, Hao-Ran Li, He-Ping Zhao, Yi Yang, Hui-Xin Zhao

**Affiliations:** ^1^ Xinjiang Key Laboratory of Special Species Conservation and Regulatory Biology, College of Life Science, Xinjiang Normal University, Urumqi, China; ^2^ Beijing Key Laboratory of Gene Resource and Molecular Development, College of Life Sciences, Beijing Normal University, Beijing, China; ^3^ Urumqi Center for Disease Control and Prevention, Urumqi, Sinkiang, China

**Keywords:** *Bacillus subtilis* BS-Z15, mycosubtilin, mouse gut microbiota, microbial community diversity, body weight

## Abstract

Our previous study reported that the metabolite of *Bacillus subtilis* BS-Z15 n-butanol site could reduce body weight gain in mice, showing anti-obesity effects. This study further demonstrated the effects of purified mycosubtilin on the body weight gain and gut microbiota of Kunming mice. 30 male Kunming white mice were randomly selected into three groups at four weeks of age: Control (group A), gavage-administered with saline daily; n-butanol site treated (group B), gavage-administered with n-butanol site metabolites (90 mg/kg); and mycosubtilin treated (group C), gavage-administered with mycosubtilin (6 mg/kg). Different groups’ gut microbiota compositions were analyzed using high-throughput sequencing technology. The results showed that the effects of mycosubtilin and n-butanol sites were consistent; the treatment groups showed a significant reduction in body weight gain as well as triglyceride and low-density lipoprotein contents as compared to the control group (P <0.05). Moreover, the size and contents of epididymal adipocytes were also significantly reduced (P <0.05), and the gavage administration of mycosubtilin could significantly increase the expression levels of lipolysis gene adipose triglyceride lipase (ATGL) in the mice liver. Mycosubtilin and n-butanol site treatments could significantly alter the composition of the gut microbiota as compared to the physiologic saline treatment. *B. subtilis* BS-Z15 metabolites may regulate weight gain primarily by mycosubtilin, according to these findings. The mycosubtilin might reduce weight gain in mice by regulating lipid metabolism, thereby reducing fat accumulation and altering the composition of gut microbiota.

## 1 Introduction

Obesity, a global health problem, is a chronic metabolic disorder, in which, the excessive production of fat occurs due to excess energy stored in the body, leading to a body mass index (BMI) of more than 30% (Apovian and Mechanick, 2013; Schetz et al., 2019). Obesity can trigger various defects in organ systems, including inflammatory responses and an imbalance of gut microbial homeostasis, which can also result in cardiovascular disease (Koliaki et al., 2019), diabetes (Shai et al., 2006), stroke (Skinner and Skelton, 2014), and some types of cancer (Winter et al., 2008). Maintaining the homeostasis of adipose tissues by regulating the adipocyte number and lipid contents are considered an effective treatment strategy for obesity (Tajbakhsh et al., 2022). In recent years, regulating gut microbiota in obesity has emerged as a potentially effective target for its treatment (Patterson et al., 2016; Liu et al., 2017). According to studies an imbalance in the abundance of specific gut microbial species might be related to the occurrence of obesity (Ridaura et al., 2013; Cerdó et al., 2019) Moreover, gut microbiota can decrease fat accumulation by stimulating fatty acid oxidation, improve insulin resistance levels in obese mice (Yang et al., 2021), enhance body immune regulation (Gu et al., 2021), and improve antioxidant capacity (Wei et al., 2021). The composition of gut microbiota can be improved with probiotics (Piccioni et al., 2022; Yanpeng et al., 2022). Moreover, studies have shown that probiotics and their metabolites have anti-obesity potential, and their addition to the diet could reduce body weight and fat pad mass in rats (Kang et al., 2010).

*B. subtilis*, a potential probiotic, can also produce a variety of biologically active lipopeptides, mainly including surface activators, iturins, fengycins, etc. (Kaspar et al., 2019). Surfactin lipopeptide produced by *B. subtilis* KLP2015 reduces Steapsin and porcine pancreatic lipase activity (Meena et al., 2018). A recent study showed that *B. subtilis* could produce fenoxin, which could eliminate *Staphylococcus aureus* by interfering with the population-sensing signals (Piewngam et al., 2018). Furthermore, it was found that feeding *Bacillus subtilis* metabolite 1-deoxynorgestimycin to obese mice could regulate the expression of hepatic lipid metabolism genes (Do et al., 2015). *B. subtilis* SPB1 could produce lipopeptide substances, which showed anti-obesity effects by inhibiting lipid digestion and liver-dysfunction enzymes (Zouari et al., 2016).

In our previous study, the therapy of mice with *B. subtilis* BS-Z15 or its fermentation products by the gavage administration of n-butanol extract showed no significant damage to the internal organs of mice (Qian-Qian et al., 2018) and activated the humoral and cellular immunity (ZY et al., 2020). At the same time, these treatments ameliorated obesity by regulating the comparative abundances of *Bacteroidetes* and *Firmicutes* in the small intestine of mice (Yang et al., 2023). High-performance liquid chromatography (HPLC) The n-butanol extract of *B. subtilis* BS-Z15 fermentation metabolite was analyzed using HPLC, which showed that the metabolite was mycosubtilin (Lin et al., 2022). Mycosubtilin is a homologue of the Icariin class (Leenders et al., 1999). However, the studies, investigating the effects of mycosubtilin on lipid decomposition and lipid synthesis, are limited. It has not been demonstrated that *B. subtilis* BS-Z15 or its fermentation products regulate mouse body weight gain through mycosubtilin. This study was conducted, Kunming mice were treated with purified mycosubtilin by gavage administration to determine if it affects body weight gain in mice. The mechanism of action of mycosubtilin was explored through its effects on the composition of mouse intestinal flora and the regulation of the regulation of lipid metabolism-related gene expression levels in mice.

## 2 Materials and methods

### 2.1 Experimental strain culture and preparation of mycosubtilin

This strain of *B. subtilis* BS-Z15 was isolated from native soil and preserved by Xinjiang Normal University’s Laboratory of Applied Biochemistry. *B. subtilis* BS-Z15 was cultured in beef paste liquid medium at 37°C for 18 h in a shaking incubator. The ratios of culture medium ingredients are in **Supplementary Table S1**.

The extraction method of then-butanol part of the metabolites was based on Zhao et al. (Qian-Qian et al., 2018). Briefly, Liquid fermentation culture of *B. subtilis* BS-Z15 was centrifuged at 9000g for 15min to obtain sterile solution, precipitated with hydrochloric acid for 12h and continued centrifugation, and collect the precipitate dissolved in water, then have n-butanol extraction to obtain BS-Z15 antifungal crude extract and volatilize the liquid phase to collect the solid and weigh. The dried metabolite was fully dissolved in dimethyl sulfoxide (DMSO) and microporous filter membranes with pores of 0.2 mm are used to remove impurities. Mycosubtilin was purified using HPLC as described by Lin et al. (Lin et al., 2022). The elution procedure is provided in **Supplementary Table S2**.

The isolated and purified mycosubtilin was weighed and compared to the n-butanol extracts to determine the mycosubtilin content in n-butanol extracts.

### 2.2 Experimental animals

Four-week-old Kunming white male mice, weighing 22–25 g. Animals were raised in accordance with the conditions at the Xinjiang Medical University Animal Experiment Center.

### 2.3 Experimental animal grouping and handling

Normal feeding for 7 days to acclimatize to the new environment, a total of 30 mice were randomly selected into 3 groups: Blank control group (Group A), gavage-administered with saline (0.5 mL per day); N-butanol site control group (Group B), gavage-administered n-butanol crude extract metabolite (a dose of 90 mg/kg per day); and mycosubtilin treated group (Group C), gavage-administered with mycosubtilin (a dose of 6 mg/kg). Drug treatment for 14 days in each group. The gavage administration dose of mycosubtilin was determined by quantifying its content in the dose of n-butanol site metabolites, having a significant modulating effect on the body weight of mice as described previously (Yang et al., 2023). Water and food were sufficient during the experiment, and mice were fed freely. The mouse feed was purchased from Xinjiang Tianyifeng biotechnology co, and its main ingredients are shown in **Supplementary Table S4**. The body weight of the mice was weighed daily at 17:30.

### 2.4 Mouse sample collection and storage

Mice were transferred to new clean cages 10 hours prior to euthanasia to stop them from eating, and they were weighed and intraperitoneally injected with sodium barbiturate for anesthetization. Sampling of blood specimens from their ventral vein and then sacrificed immediately by cervical dislocation. Their livers, spleens, hearts, and epididymal and intestinal fat tissues were removed by dissecting their abdominal cavities on the bench. Blood was rinsed with saline, which was absorbed using filter paper and weighed and recorded. The epididymal fat rate, intestinal fat rate, and the corresponding organ indices were calculated. The Fat rate=(Fat weight/Mouse body weight)*100. The epididymal fat tissues were stored in 10% neutral phosphate buffered formalin for 24 h and then observed after hematoxylin-eosin (H&E) staining (Cardiff et al., 2014). The remaining organs were snap-frozen in liquid nitrogen and saved in a –80°C refrigerator. Blood samples were rested at 4°C for 3h and centrifuged at 3000g for 10 min. 50-uL of plasma was stored at -80°C in the refrigerator after quick-freezing with liquid nitrogen.

### 2.5 Detection of triglyceride and total cholesterol concentration in the liver and low-density lipoprotein and high-density lipoprotein concentration in the plasma

Mouse liver tissue was weighed (0.5 g) and mechanically homogenized in 4.5 mL anhydrous ethanol. The homogenized solution was centrifuged at 3000 g for 10 min. A supernatant of 50-µL was collected and TC (Richmond, 1973) and TG (Sullivan et al., 1985) concentration were determined. A 50-µL frozen serum sample was taken and melted on ice for the detection of LDL (Rifai et al., 1998) and HDL (Schectman et al., 1996) concentration. The assay was performed using enzyme-labeled instrument, and the method steps were strictly in accordance with the instructions of the Nanjing built kit (http://www.njjcbio.com).

### 2.6 Detection of lipid metabolism- and lipid synthesis-related genes expression levels in mice

A 0.3-g mouse liver tissue was taken for RNA extraction. Extracted total RNA from liver tissues using liquid nitrogen grinding using TRIzol reagent, 1 µl of total RNA solution per sample was taken for quantification and integrity analysis using a micro-nucleic acid detector and then reverse-transcribed into cDNA using a Tiangen reverse transcription kit. The mRNA expression levels of lipid synthesis-related genes, including peroxisome proliferator-activated receptor-alpha (*PPARα*) and transcription factor sterol regulatory element binding protein*-*1 (*SREBP-1*), and lipid metabolism-related genes, including hormone-sensitive lipase (*HSL*) and adipose triglyceride lipase (*ATGL*). The fluorescence real-time quantitative PCR (RT-qPCR) method was used to determine the results, the PCR reaction conditions were as follows: initially denaturing at 95°C for 5 min, then annealing at 58°C for 30 s and extending at 72°C for 30 s; and finally extending at 4°C for 10 min. The specific primers were designed according to the gene sequences (**Supplementary Table S3**), and Bioengineering (Shanghai) Co. synthesized the primers.

### 2.7 Detection of gut microbiota after mycosubtilin treatment

The small intestinal contents were collected and stored at –80°C in a refrigerator. Bacterial DNA was extracted from the small intestinal contents. The V3-V4 region of the *16S rDNA* gene was amplified using PCR. The PCR amplicons were quantified, and a pool was created to construct the MiSeq library for MiSeq sequencing. Paired-end raw sequence reads were obtained. The overlapping sequences were spliced followed by quality control and filtering of sequences. As a result of differentiating the samples, an OTU (operational taxonomic unit) clustering analysis was conducted. This allowed the analysis of different diversity indices based on OTU sets and detected the depth of high-throughput sequencing. The composition of gut microbiota was statistically analyzed at different taxonomic levels, referring to the corresponding taxonomic information. Based on the above analysis, the information on the composition of intestinal flora in the mycosubtilin treatment group was visualized by multivariate analysis and Student’s t-test. Bioinformatics analyses were performed on the high-quality sequencing data, and functional prediction of the 16S amplicon sequencing results was performed using the Phylogenetic Investigation of Communities by Reconstruction of Unobserved States (PICRUSt) software package, in which the OTU abundance table was standardized by PICRUSt. The OTU abundance table was standardized by PICRUSt, and then the COG family information and KEGG Ortholog (KO) information corresponding to each OTU were obtained from the greengene id of each OTU; the abundance of each COG and the KO abundance were calculated. Based on the information of COG database, the description information of each COG, as well as its functional information, can be resolved from eggNOG database, so as to obtain the functional abundance spectrum; based on the information of KEGG database, the KO, Pathway, and EC information can be obtained, and the abundance of each functional category can be calculated based on the OTU abundance.

### 2.8 Statistical analyses

SPSS 19 software was used to perform the statistical analyses. The metric data were mainly expressed as mean ± standard deviation. Differences among multiple groups were analyzed using one-way analysis of variance (ANOVA). Different lowercase letters represent significant differences at *P <*0.05. In addition, graphs were plotted with Origin 8.5. OTU clustering was performed at a 97% similarity level using USEARCH software. The obtained OTUs were taxonomically annotated based on the silva138/16s_bacteria (https://www.arb-silva.de/) taxonomic databases. Using R language tools, community structure maps were plotted at the animal taxonomic level. Information on the analytical software and database used for the analysis and the version number is in **Supplementary Table S5**. Majorbio Cloud Platform was used to analyze the data (www.majorbio.com).

## 3 Results

### 3.1 Purification and dose determination of mycosubtilin in n-butanol extract

**Figure 1:**
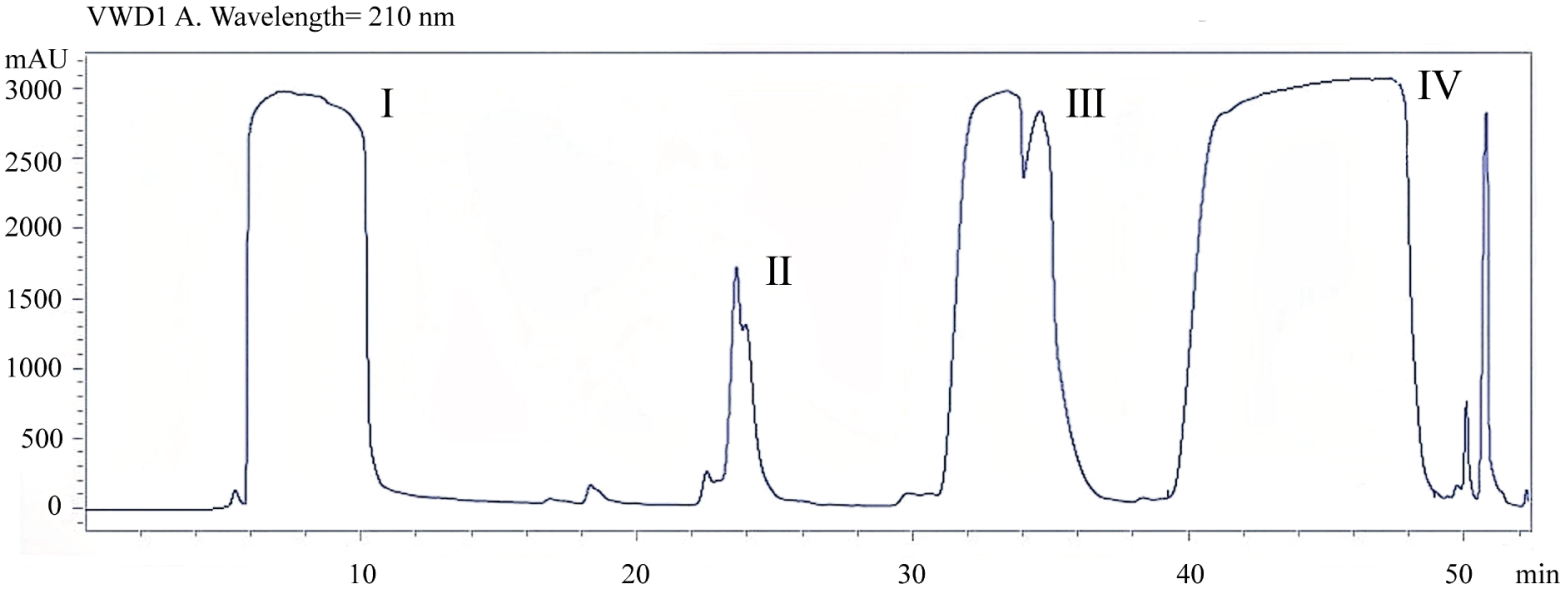
Determination of mycosubtilin content in crude extracts by n-butanol extraction.

In order to determine the concentration of mycosubtilin in the n-butanol extract of *B. subtilis* BS-Z15, the metabolites in the DMSO-dissolved n-butanol site were detected using HPLC separation. Mycosubtilin was characterized as peak IV with a peak time of 40–48 min. The concentration of mobile phase acetonitrile was 50%, which was similar to that used by Lin et al. (**Figure 1**). A total of 20 mg of mycosubtilin was isolated and purified from 300 mg of the n-butanol site. According to a previous study, 90 mg/kg of n-butanol site metabolite could significantly affect the body weight gain of mice (Yang et al., 2023).

### 3.2 Effects of mycosubtilin on the weight gain of mice

**Figure 2:**
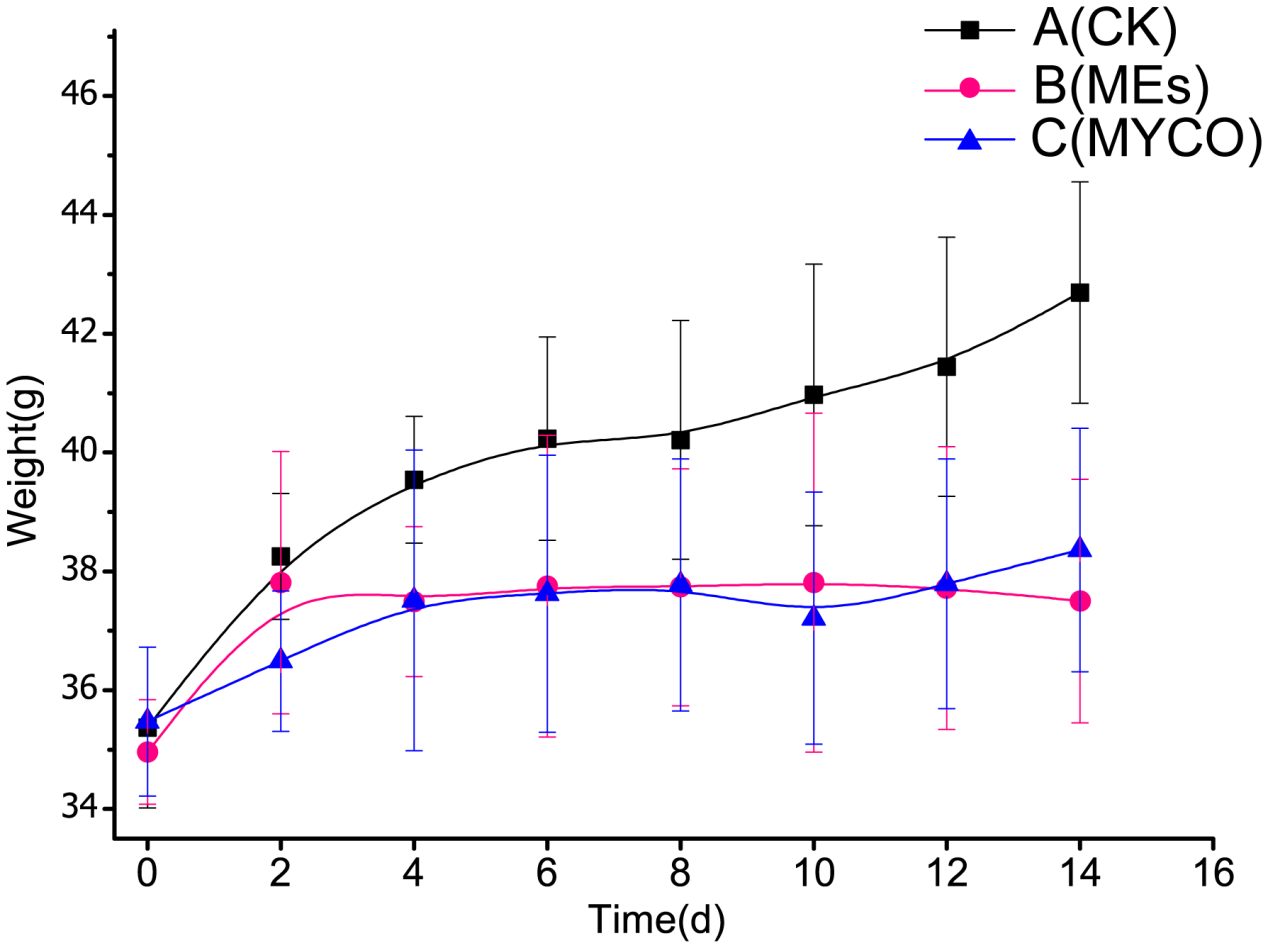
Effect of mycosubtilin on body weight gain in mice A(CK): blank control group, B(MEs): n-butanol site control group, C(MYCO): mycosubtilin fumigatus-treated group, unit g, n=6.

Current research suggests that, gavage of *B. subtilis* BS-Z15 and its metabolites were consistent with the effects of pregavage of *B. subtilis* BS-Z15 on slowing the body weight of mice and did not affect the amount of food they ate, so the effect of diet of mice on the results was not considered in this case. Both gavage of *B. subtilis* BS-Z15 n-butanol site metabolites and purified mycosubtilin reduced body weight gain in mice compared to controls; the body weights of mice in groups B and C were significantly lower as compared to the control group mice from day 4 and lasting until day 14 (*P <*0.05) (**Figure 2**). The heart, kidney, and spleen indices of the mice at the end of the experiment were also observed, showing no significant differences in groups B and C as compared to group A. However, at the end of the experiment, the liver index of mice in group B was significantly lower than that in group A (*P <*0.05). Moreover, the liver indices in group C tended to decrease as compared to group A; however, the difference was insignificant (**Table 1**).

**Table 1:** Effect of mycosubtilin resistance on organ indices in mice.

Viscera	A	B	C
Cardiac	4.57 ± 0.23^a^	4.24 ± 0.27^a^	4.20 ± 0.38^a^
Kidney	16.39 ± 1.44^a^	15.93 ± 1.39^a^	16.08 ± 0.91^a^
Liver	56.17 ± 2.16^a^	52.12 ± 1.33^b^	54.00 ± 1.24^ab^
Spleen	2.73 ± 0.46^a^	2.74 ± 0.61^a^	3.28 ± 0.43^a^

A(CK): blank control group, B(MEs): n-butanol site control group, C(MYCO): mycosubtilin fumigatus-treated group, the different superscript letters denote statistical difference between groups, unit mg/g, n=6, p<0.05.

### 3.3 Effects of mycosubtilin on adipose tissues in mice

**Figure 3:**
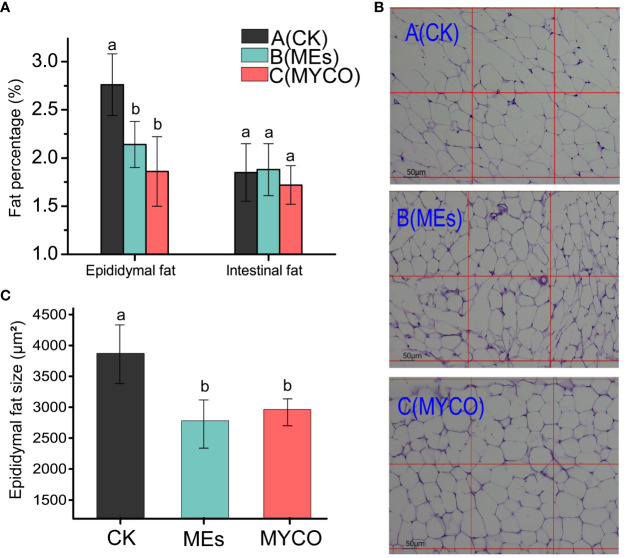
Effects of mycosubtilin on white fat weight and fat morphology in mice **(A)** Changes in epididymal adiposity and intestinal adiposity (unit mg/g); **(B)** Mouse epididymal fat after different treatments was stained by H&E and photographed at 100x magnification (scale bar = 50 μm); **(C)** Statistical analysis of epididymal fat cell size, the different superscript letters denote statistical difference between groups, n=6, p<0.05.

The present study found, after gavage of *B. subtilis* BS-Z15 n-butanol site metabolite and purified mycosubtilin in mice, the obesity rate of epididymis in groups B and C was remarkably less than that in group A (*P <*0.05), while the percentage of intestinal fat had no significant differences in the treatment and untreated groups (**Figure 3A**). Investigating the H&E-stained sections of epididymal fat tissues further revealed that the mean size of epididymal adipocytes decreased significantly in treatment groups B and C compared with control group A (**Figures 3B, C**) (*P <*0.05). These results suggested that the metabolites of mycosubtilin and n-butanol sites could reduce epididymal fats in mice by reducing the size of epididymal adipocytes.

### 3.4 Effects of mycosubtilin on liver and serum adipokines in mice

**Figure 4:**
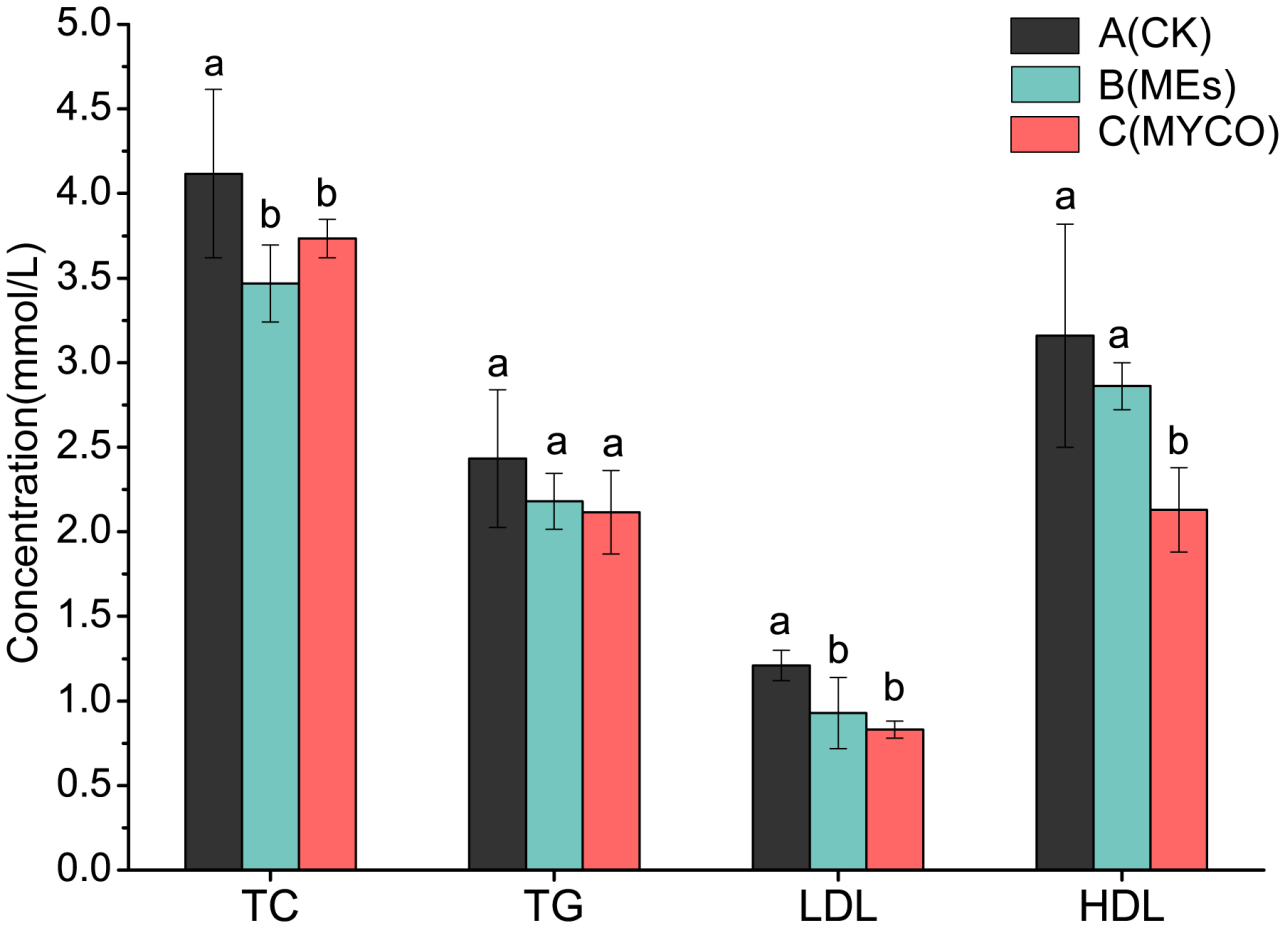
Effects of mycosubtilin on TC, TG, HDL and LDL in mice. The different superscript letters denote statistical difference between groups, n=6, p<0.05.

At the end of the gavage treatment, the gavage administration of *B. subtilis* BS-Z15 n-butanol site metabolite and purified mycosubtilin had no significant effect on liver TC concentration in mice in groups B and C as compared with control group A. Compared with the control group A, groups B and C had significantly lower liver TG concentration and plasma LDL levels (*P <*0.05). However, there was no significant change in plasma HDL levels in group B mice and a significant decrease in plasma HDL in group C mice compared to control group A (*P <*0.05) (**Figure 4**).

### 3.5 Effects of mycosubtilin on the expression levels of lipid metabolism-related genes in mice

**Figure 5:**
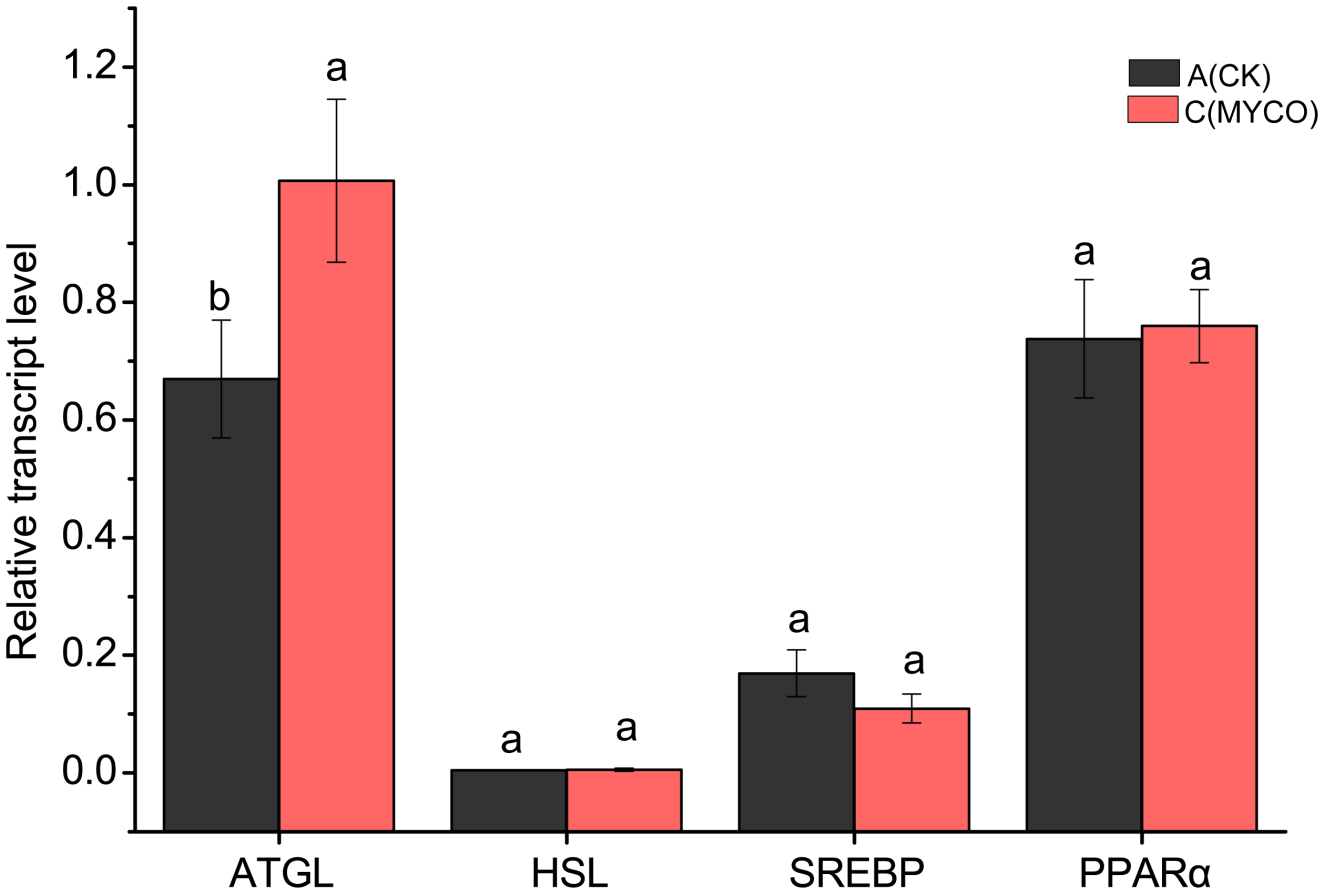
Effect of mycosubtilin on liver lipid metabolism genes in mice. The different superscript letters denote statistical difference between groups, n=6, p<0.05.

To further investigate the role of mycosubtilin on adipogenesis, the mRNA expression levels of key liver lipid metabolism-related genes were detected using RT-qPCR. The results revealed that mycosubtilin treatment could noticeable enhancement the expression levels of lipolysis gene *ATGL* (*P <*0.05) in group C and decrease that of lipolysis gene *SREBP*. On the other side, the mycosubtilin treatment had no obviously impact on the expression levels of *HSL* and *PPARa* (**Figure 5**).

### 3.6 Effects of mycosubtilin on the gut microbiota of mice

**Figure 6:**
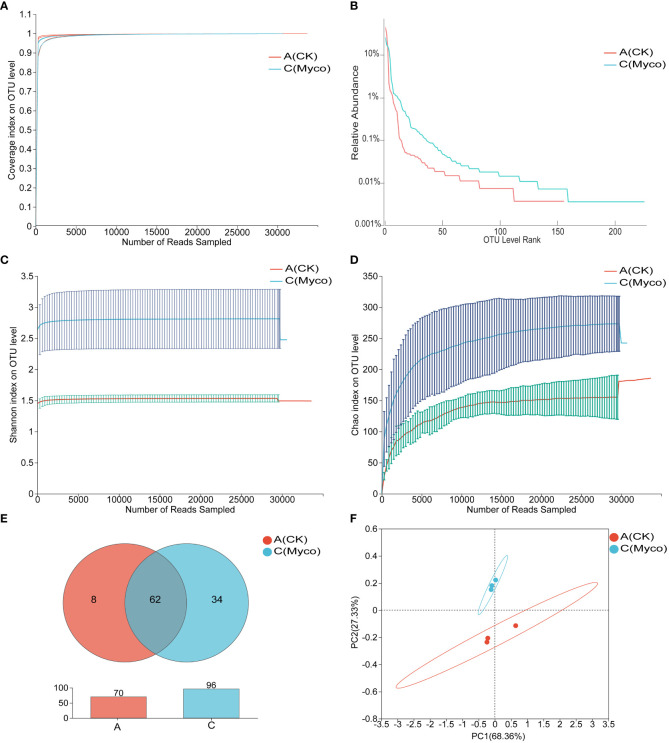
Effect of mycosubtilin on species diversity of intestinal flora in mice **(A)** Coverage curves of OTU level; **(B)** Rank-Abundance curves of OTU level; **(C)** Shannon's index of OTU level; **(D)** Chao's index of OTU level; **(E)** Venn diagrams of genus level; **(F)** PCoA analysis at the OTU level, n=3.

Based on bacterial *16S rRNA* gene sequencing, a total of 208,780 valid sequences, including 88,621,609 bases, were obtained for all the groups. The average number of valid sequences in Groups A and C are 35,515 and 34,078.33, respectively, and the number of bases is 15,066,529 and 14,474,007, respectively. Three samples per group, the sequencing coverage was above 0.99, which suggest that the probability of undetected sequences in the specimen is low and well-reflected the total gut microbiota in each sample (**Figure 6A**). Rank-Abundance curves were used to compare the differences in species composition of the mouse gut microbiome after MYCO treatment. The results showed that the shapes of the curves for the bacterial communities were roughly similar in the different groups, but deviated from the same abundance thresholds (**Figure 6B**). Shannon and chao indices were found to increase the diversity of the mouse gut community and community richness after MYCO treatment (**Figures 6C, D**). The number of shared taxa at the genus level between the groups are shown in Venn diagram. An average of 96 genera were detected in mycosubtilin-treated group C, and 70 genera were detected in group A of the blank control (**Figure 6E**). The principal coordinates analysis (PCoA) was executed to explore the dissimilarity among microbial communities obtained from different treatment of mouse small intestine. Mycosubtilin treated group C explained 68.36% and 27.33% of bacterial variation by PCoA1 and PCoA2 compared to blank control group A, respectively. (**Figure 6F**). The mycosubtilin-treated group C showed a significantly higher abundance of gut microbiota in mice as compared to that in the control group.

**Figure 7:**
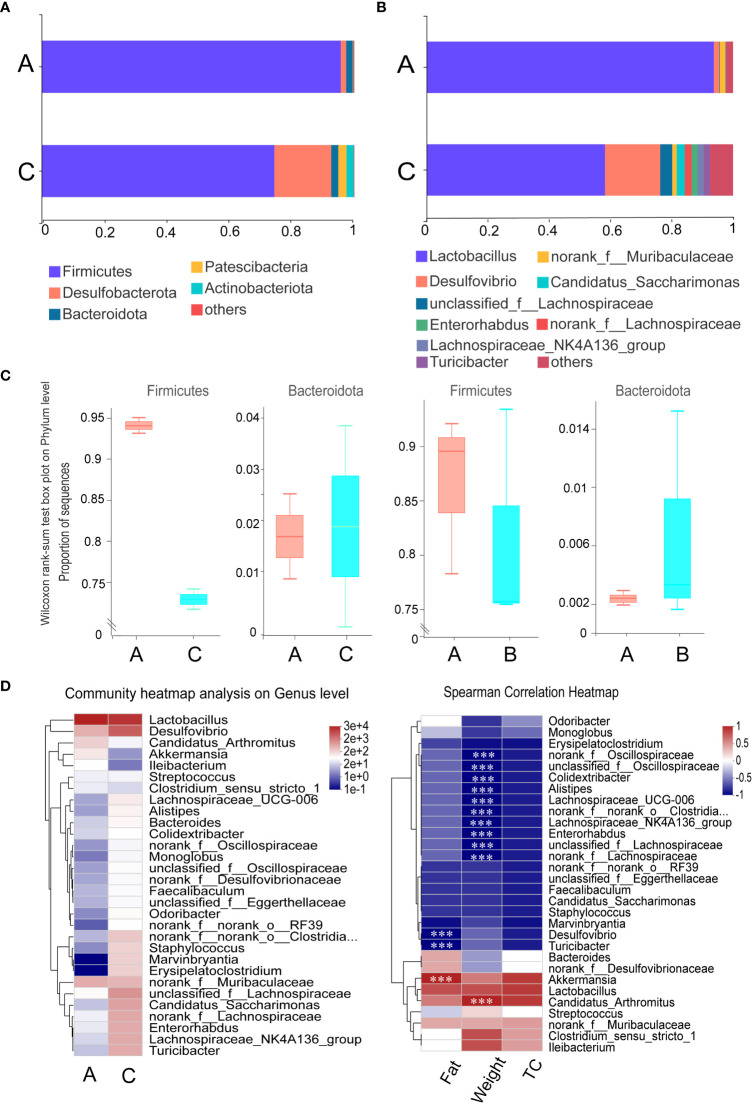
Effect of mycosubtilin on bacterial community composition in the small intestine of mice: **(A)** Histogram of relative abundance of gut flora at the phylum level; **(B)** Histogram of relative abundance of gut flora at the genus level; **(C)** Comparison of abundance of the phylum Firmicutes and Bacteroides after mycosubtilin treatment and n-butanol site treatment; **(D)** Community heatmap analysis on Genus level & Heatmap of Spearman correlation analysis, n=3, *** P ≤ 0.001.

At the phylum level, *Firmicutes* were the most dominant group of microbes based on relative abundances. The gavage administration of mice with mycosubtilin reduced the relative abundance of *Firmicutes* (A: 95.65% *vs.* C: 74.40%) and increased that of *Bacteroidota* (A:2.04% *vs.* C:2.26%) as compared to the blank group A. Those results were consistent with those from previous studies investigating *B. subtilis* BS-Z15 metabolites’ n-butanol effects (**Figures 7A–C**). The mycosubtilin treatment could increase the relative abundance of *Desulfobacterota* (A: 1.73% *vs.* C: 16.41%). The relative abundance of Lactobacillus decreased with mycosubtilin at the genus level (A:93.76% vs. C:58.23%) and increased *Desulfovibrio* (A: 1.71% vs. C: 18.03%), *Lachnospiraceae_NK4A136_group* (A:0.06%, C:2.05%) abundance, thereby altering the composition of gut microbiota (**Supplementary Figure 1**). Heatmap and Spearman’s correlation analyses of the top 30 genera in terms of genus-level abundance showed that *Lactobacillus* and *Candidatus_Arthromitus* were positively correlated with Fat, TC and body weight, and *Lachnospiraceae_NK4A136_group* and *Desulfovibrio* were negatively correlated with Fat, TC and body weight (**Figure 7D**).

**Figure 8:**
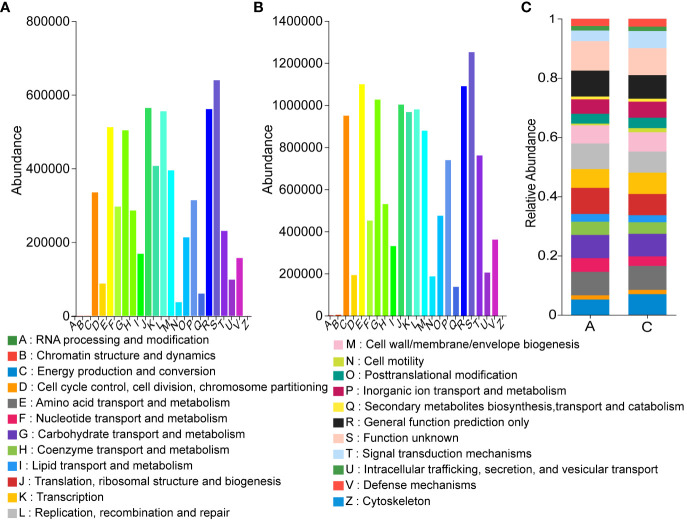
Effect of mycosubtilin predicted by PICRUSt on the relative abundance of COG functional classification **(A)** Group A COG based function abundances of inoculum; **(B)** Group C COG based function abundances of inoculum; **(C)** Relative abundances of functions based on COG classification, n=3.

Calculation of abundance profiles for each functional category by PICRUSt on OTU abundance revealed that the predicted relative abundance of COG functional compositions was more similar in all samples compared to the species composition, with Amino acid transport and metabolism, Carbohydrate transport and metabolism. Amino acid transport and metabolism, Carbohydrate transport and metabolism, and Energy production and conversion all showed high abundance in different groups. However, the function with the highest abundance was set as unknown, indicating that the COG database was not rich enough to fully reveal the functions of the microbial community (**Figures 8A, B**). Comparison of KEGG database showed that Metabolism was the most abundant at Level 1 and Membrane Transport was the most abundant at Level 2 (**Supplementary Table S4**). For comparison, we further analyzed the COG-based functions in all samples showing the relative abundance of different functions, where the top three up-regulated relative expression abundance of functions in the treatment group compared to the control group were: Energy production and conversion (A: 5.22%, C: 6.97%), Signal transduction mechanisms (A: 3.59%, C: 5.59%), Inorganic ion transport and metabolism (A: 4.89%, C: 5.43%), The top three functional relative expression abundances were down-regulated, respectively: Replication, recombination and repair (A: 8.65%, C: 7.19%); Translation, ribosomal structure and biogenesis (A: 8.79%, C: 7.36%); Nucleotide transport and metabolism (A: 4.62%, C: 3.32%) (**Figure 8C**).

## 4 Discussion

The excessive accumulation of adipocytes in the subcutis and viscera is a major cause of obesity (Gesta et al., 2007). Excessive fat formation might lead to an increased number of adipocytes and fat volume (Camp et al., 2002) and is the main determinant of overweight and obesity. *B. subtilis* BS-Z15 and its n-butanol site metabolites have been shown to decrease in epididymal fat rate in mice in a previous study (Yang et al., 2023). The present study showed that the gavage administration of mice with *B. subtilis* BS-Z15 metabolite mycosubtilin could significantly reduce body weight gain and epididymal adiposity as well as the mean epididymal adipocyte size in mice. This showed that mycosubtilin had the same effect as that of n-butanol site metabolites, suggesting that mycosubtilin could reduce body weight gain in mice by reducing fat accumulation. As a white fat, epididymal fat can not only store energy in the form of TG but can also act as an endocrine organ, secreting a variety of adipocytic factors, such as lipocalin and tumor necrosis factor et al. (Sotak et al., 2021). The mice’s liver and blood tests revealed that the n-butanol sites and mycosubtilin could significantly reduce the TG and LDL concentration. These results were also similar to those reported in previous studies that lipopeptides could regulate the TC concentration by inhibiting serum pancreatic lipase activity, thereby showing anti-obesity effects (Shao et al., 2015; El Bekay et al., 2016). Another study showed that lipopeptide substances produced by *B. subtilis* SPB1 could achieve anti-obesity effects by inhibiting lipid digestive enzymes and liver dysfunction enzymes (Zouari et al., 2016). The effects of mycosubtilin, a lipopeptide homolog of ecdysone, on regulating lipid metabolism and lipid synthesis-related protease activity require further exploration. This indicated that mycosubtilin could reduce obesity in mice by regulating lipid factors, thereby showing an anti-obesity activity.

Lipid accumulation is regulated by many genes. *SREBPs*, which are involved in cholesterol and lipid metabolism, are important transcription factors in hepatocytes (Oliva et al., 2013). ATGL initiates lipid hydrolysis and is the key enzyme, catalyzing lipid reserve in mammalian adipose tissues (Iftikhar et al., 2021). In this study, mycosubtilin could significantly upregulate the lipolytic *ATGL* gene, suggesting that mycosubtilin might regulate lipid metabolism in mice by regulating the expression levels of lipolytic genes, thereby reducing the adiposity in mice. This is also consistent with that feeding the *B. subtilis* metabolite 1-deoxynorgamycin to mice modulates the effects of hepatic lipid metabolism gene expression (Do et al., 2015). Simultaneously, mycosubtilin could also reduce the expression levels of lipid synthesis *SREBP* gene while having no effects on those of *HSL* and *PPAR*. Mycosubtilin might not only regulate lipid catabolism but also affect lipid synthesis; therefore, the lipid metabolism-related pathways, regulated by mycosubtilin, require further in-depth studies.

Gut microbiota is an important component involved in human metabolism by supplying the metabolic process with enzymes and energy. In addition, the gut microbial barrier protects the organism against foreign bacterial infections (Abenavoli et al., 2019; Wang et al., 2022). Gut microbiota can induce obesity mainly by providing excess energy to the host and producing inflammatory metabolites (Xu et al., 2016). Due to the diversity of gut microbiota, regulating its composition might have an anti-obesity effect (Cani et al., 2007; Gomes et al., 2020; Geng et al., 2022). Therefore, decreasing the abundance of pro-obesity microbiota and increasing that of anti-obesity microbiota in the host’s gut might alleviate obesity. Moreover, the analysis of gut microbiota revealed that both mycosubtilin and n-butanol sites could reduce the abundance of *Firmicutes* and increase that of *Bacteroidota*; these results were consistent with the previous research has shown that *B. subtilis* BS-Z15 alters gut microbiota and changes the abundance of phyla to reduce weight gain (Stojanov et al., 2020). This suggested that mycosubtilin might reduce weight gain by modulating the composition of gut microbiota. The gut microbiota has been reported to inhibit obesity in several ways, such as reducing intestinal mucosal permeability, enhancing intestinal mucosal integrity, elevating tight junction protein levels, reducing insulin resistance, and promoting the browning of white fat (Plovier et al., 2017; Yoon et al., 2021). Gavage of *B. subtilis* BS-Z15 n-butanol site metabolites and mycosubtilin significantly increased bacterial abundance and increased *Lachnospiraceae_NK4A136_group* abundance, altering the colony structure, and one study found that obesity-related metrics were negatively correlated with the relative abundance of *Lachnospiraceae_NK4A136_group* (Li et al., 2022). Less weight gain in mice was strongly correlated with these changes in gut flora composition. Mycosubtilin and n-butanol site metabolites did not affect gut microbiota composition in the same way, which may be due to the effects of other components in the n-butanol site. The current study showed that mycosubtilin could increase the abundance of *Desulfovibrio*. A previous study also reported that *Desulfovibrio* was a potent acetic acid generator, which could reduce liver steatosis by regulating liver adipokine metabolism in mice by producing acetic acid (Hong et al., 2021). This suggested that mycosubtilin could further regulate its metabolites by modulating gut microbiota. The accuracy of functional gene prediction of gut flora by PICRUSt software was 85%-90% (Yin and Wang, 2020). Most of the genes encoded by gut microbes in mycosubtilin -treated mice were found to be metabolism-related by comparison with the KEGG database, and functional prediction of the 16S amplicon sequencing results by PICRUSt also revealed that mycosubtilin treatment had an effect on energy production and conversion and inorganic ion transport and metabolism, etc., Mei et al. analyzed the gut microbiota through PICRUSt population may influence the cause of inflammation and metabolism in the host due to altered functional expression (Mei et al., 2021). At the same time, the mechanism of mycosubtilin, regulating the intestinal environment, was not clear because gut microbiota can metabolize a variety of compounds with biological functions *in-vivo*, this could play a crucial role in regulating distal organ metabolism. Therefore, the changes in the intestinal microenvironment of mice and the effects of mycosubtilin on regulating body weight gain after mycosubtilin treatment should be further investigated; this will be the direction of our group’s future research. These findings also provided preliminary evidence that mycosubtilin was the main *B. subtilis* BS-Z15 metabolite, which could reduce weight gain in mice and had potential anti-obesity effects.

There were some limitations to this study, which should be considered. First, although the grouping was randomized, a relatively small sample size was used, and mice may differ in their gut microbiota abundance depending on their individual differences, affecting the assessment of the small gut microbiota. Second, although the proportion of mycosubtilin in the metabolites of the n-butanol fraction was calculated in this study, the effects of other metabolites in the crude extract on body weight gain in mice were not investigated. Therefore, future synergistic effects of other fractions should be compared with that of mycosubtilin. Finally expanding mouse rearing and improving environmental factors should also be considered to study the effects. Due to these limitations, the correlations between mycosubtilin-resistant gut microbiota and lipid metabolism pathways require in-depth studies.

In conclusion, the effects of gavage administration of n-butanol sites and mycosubtilin on gut microbiota composition and body weight gain were investigated. Its gavage administration could also significantly increase the abundance and diversity of gut microbiota at genus levels after mycosubtilin administration significantly reduced weight gain in mice in the study, increase the abundance of phylum *Bacteroidetes*, and decrease that of phylum *Firmicutes*. These results were consistent with the effects of n-butanol sites on body weight gain in mice, suggesting that mycosubtilin was the main effector in regulating body weight gain in mice. Further research will be needed, in detail the correlations between weight gain and gut microbiota composition and the underlying mechanisms, this will also be the focus of our future work. This study provided a basis for the application of mycosubtilin in regulating body weight gain in animals.

## Data availability statement

The data supporting the findings of this study have been deposited into the CNGB Sequence Archive (CNSA) of the China National GeneBank DataBase (CNGBdb) under the accession number CNP0005267 (https://db.cngb.org/).

## Ethics statement

The animal study was approved by Animal protocols approval: Xinjiang Medical University, SYXK(Xin) 2018-0003. The study was conducted in accordance with the local legislation and institutional requirements.

## Author contributions

JY: Funding acquisition, Project administration, Writing – original draft, Writing – review & editing. JJ-Z: Conceptualization, Investigation, Visualization, Writing – review & editing. J-QY: Data curation, Methodology, Software, Visualization, Writing – review & editing. M-YM: Data curation, Project administration, Writing – review & editing. HL: Data curation, Visualization, Writing – review & editing. J-YC: Data curation, Visualization, Writing – review & editing. X-YC: Data curation, Methodology, Visualization, Writing – review & editing. H-RL: Data curation, Methodology, Visualization, Writing – review & editing. H-PZ: Conceptualization, Resources, Writing – review & editing. YY: Writing – review & editing. H-XZ: Funding acquisition, Investigation, Resources, Supervision, Writing – review & editing.
