# Supplementary figures and images for: Effect of Bacillus subtilis BS-Z15 metabolite mycosubtilin on body weight gain in mice
Source: Front Microbiomes. 2024 Mar 13;3:1301857. doi: 10.3389/frmbi.2024.1301857 (PMC12993509; doi:10.3389/frmbi.2024.1301857)

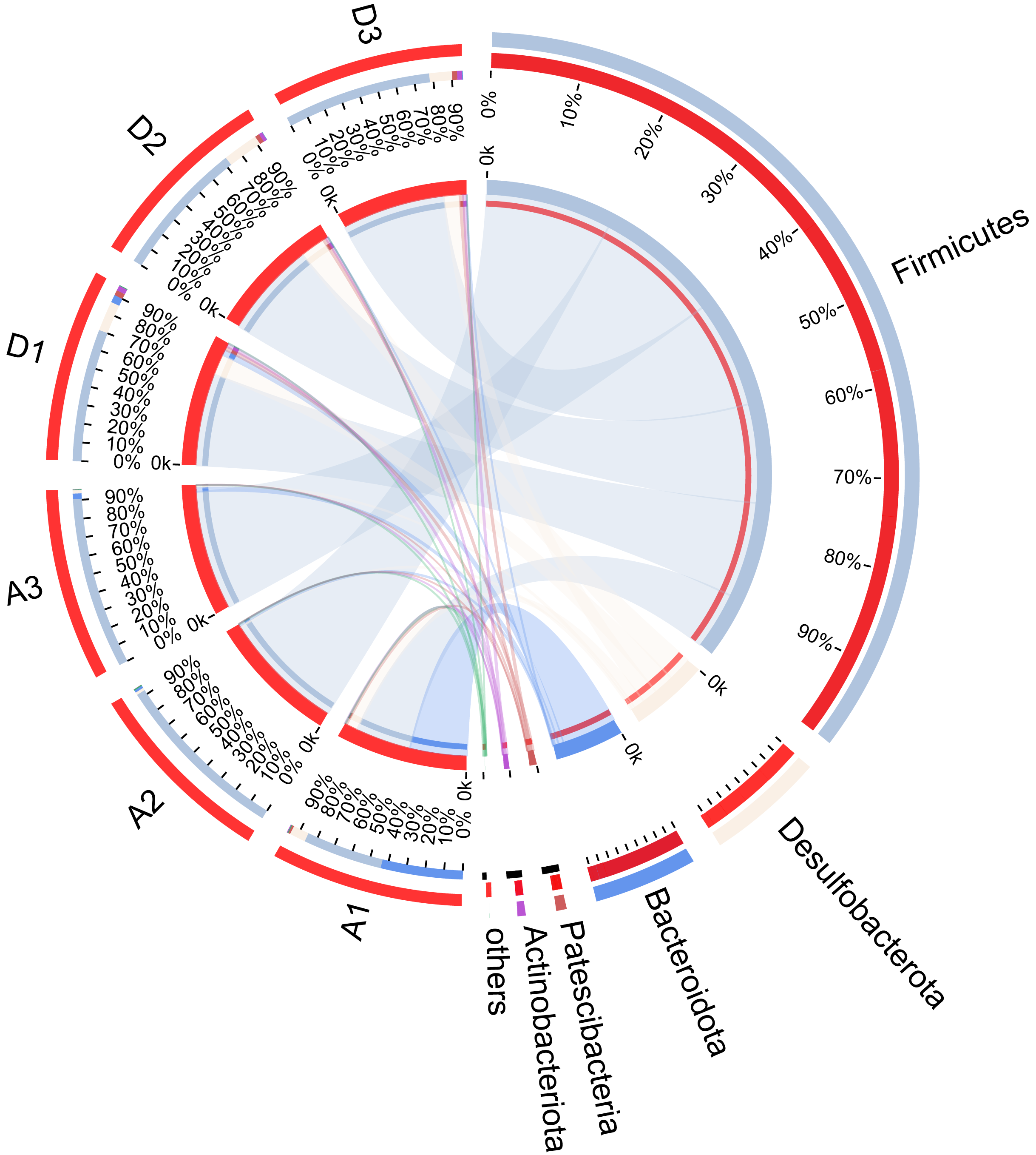

Supplement: Supplementary file 3 [file Image_1.tif]
