# Supplementary material for: Effect of Bacillus subtilis BS-Z15 metabolite mycosubtilin on body weight gain in mice
Source: Front Microbiomes. 2024 Mar 13;3:1301857. doi: 10.3389/frmbi.2024.1301857 (PMC12993509; doi:10.3389/frmbi.2024.1301857)
Supplement: Supplementary file 4 [file Table_1.docx]

| Media composition | Content(%) | Manufacturer |
| --- | --- | --- |
| Beef Extract | 0.3 | Solarbio |
| Tryptone | 1.0 | OXOID |
| NaCl | 0.5 | Tianjin Beilian |

**SI Table 1** Beef extract peptone medium composition

The culture medium components contained in 100 ML distilled water with a final pH=7 were sterilized in portions (121°C, 20 min).
