# Supplementary material for: Effect of Bacillus subtilis BS-Z15 metabolite mycosubtilin on body weight gain in mice
Source: Front Microbiomes. 2024 Mar 13;3:1301857. doi: 10.3389/frmbi.2024.1301857 (PMC12993509; doi:10.3389/frmbi.2024.1301857)
Supplement: Supplementary file 5 [file Table_2.docx]

**SI Table 2** Mobile phase elution gradients

| Time  （min） | Flow rate  (mL/min） | A（%） | B（%） |
| --- | --- | --- | --- |
| 0 | 3 | 60 | 40 |
| 30 | 3 | 50 | 50 |
| 40 | 3 | 0 | 100 |
| 60 | 3 | 0 | 100 |

Mobile phase A is water, mobile phase B is acetonitrile, UV detection light is 210 nm, and the single injection volume is 50μL.
