# Supplementary material for: Effect of Bacillus subtilis BS-Z15 metabolite mycosubtilin on body weight gain in mice
Source: Front Microbiomes. 2024 Mar 13;3:1301857. doi: 10.3389/frmbi.2024.1301857 (PMC12993509; doi:10.3389/frmbi.2024.1301857)
Supplement: Supplementary file 6 [file Table_3.docx]

**SI Table 3** Sequence-specific primers for lipid metabolism genes in mouse liver

| Primer name | Sequence (5'→3') |
| --- | --- |
| SREBP-1 | F: CAGACTCGCTGCTTCTGACA |
|  | R: GGACTGTTGGCCAAGATGGTT |
| PPARα | F: CAGTTCTGGAGGCTGGGAAG |
|  | R: CACCATCGCGACCAGATGG |
| HSL | F: ACTGAGATTGAGGTGCTGTC |
|  | R: TGGCGTTGTCTCTGGAGATG |
| ATGL | F: GТСАССААСАССAGСAТCC |
|  | R: CGAAGTCCATCTCTGTAGCC |
| β-actin | F: AGGCCCAGAGCAAGAGAGG |
|  | R: TACATGGCTGGGGTGTTGAA |
