# Supplementary material for: Effect of Bacillus subtilis BS-Z15 metabolite mycosubtilin on body weight gain in mice
Source: Front Microbiomes. 2024 Mar 13;3:1301857. doi: 10.3389/frmbi.2024.1301857 (PMC12993509; doi:10.3389/frmbi.2024.1301857)
Supplement: Supplementary file 8 [file Table_5.docx]

**SI Table 5** Information on analytical software and databases used for project analysis

| Software/  Database | Version number | Software/Database purposes | Database links |
| --- | --- | --- | --- |
| Flash | 1.2.11 | pair-end double-ended sequence splicing | https://ccb.jhu.edu/software/FLASH/index.shtml |
| Qiime | 1.9.1 | Generation of water abundance tables for each taxonomy, beta diversity distance calculation | http://qiime.org/install/index.html |
| Uparse | 11 | OTU Clustering Algorithm 1 | http://www.drive5.com/uparse/ |
| RDP Classifier | 2.13 | Sequence Classification Notes | https://sourceforge.net/projects/rdp-classifier/ |
| Usearch | 11 | OTU statistics | http://www.drive5.com/usearch/ |
| Mothur | 1.30.2 | alpha diversity analysis | https://www.mothur.org/wiki/Download_mothur |
| PICRUSt | 1.1.0 | Prediction of KEGG, COG, and Pfam functions of 16S sequences | http://picrust.github.io/picrust/ |
