# Supplementary material for: Effect of Bacillus subtilis BS-Z15 metabolite mycosubtilin on body weight gain in mice
Source: Front Microbiomes. 2024 Mar 13;3:1301857. doi: 10.3389/frmbi.2024.1301857 (PMC12993509; doi:10.3389/frmbi.2024.1301857)
Supplement: Supplementary file 9 [file Table_6.docx]

**SI Table 6** Effect of mycosubtilin on body weight gain in mice

| Day | A | B | C |
| --- | --- | --- | --- |
| 0 | 35.37±1.35^a^ | 34.96±0.88^a^ | 35.47±1.25^a^ |
| 2 | 38.25±1.06^a^ | 37.81±2.21^a^ | 36.24±1.49^a^ |
| 4 | 39.54±1.07^a^ | 37.39±2.71^b^ | 36.61±1.98^b^ |
| 6 | 40.23±1.71^a^ | 37.75±2.71^b^ | 37.21±2.12^b^ |
| 8 | 40.21±2.01^a^ | 37.73±1.99^b^ | 37.62±2.33^b^ |
| 10 | 40.97±2.20^a^ | 37.81±2.85^b^ | 37.77±2.12^b^ |
| 12 | 41.44±2.18^a^ | 37.72±2.38^b^ | 37.79±2.10^b^ |
| 14 | 42.69±1.86^a^ | 37.50±2.05^b^ | 38.36±2.05^b^ |

A: blank control group, B: n-butanol site control group, C: antimycobacterium fumigatus-treated group,The different superscript letters denote statistical difference between groups unit g, n=6, p<0.05
